# Downregulation of SPARC expression decreases gastric cancer cellular invasion and survival

**DOI:** 10.1186/1756-9966-29-59

**Published:** 2010-06-02

**Authors:** Jie Yin, Guowei Chen, Yucun Liu, Si Liu, Pengyuan Wang, Yuanlian Wan, Xin Wang, Jing Zhu, Hongqiao Gao

**Affiliations:** 1 Department of General Surgery, Peking University Fisrt Hospital, Beijing, 100034, PR China

## Abstract

**Background:**

Secreted protein acidic and rich in cysteine (SPARC) plays a key role in the development of many tissues and organ types. Aberrant SPARC expression was found in a wide variety of human cancers, contributes to tumor development. Because SPARC was found to be overexpressed in human gastric cancer tissue, we therefore to explore the expression of SPARC in gastric cancer lines and the carcinogenic mechanisms.

**Methods:**

SPARC expression was evaluated in a panel of human gastric cancer cell lines. MGC803 and HGC 27 gastric cancer cell lines expressing high level of SPARC were transiently transfected with SPARC-specific small interfering RNAs and subsequently evaluated for effects on invasion and proliferation.

**Results:**

Small interfering RNA-mediated knockdown of SPARC in MGC803 and HGC 27 gastric cancer cells dramatically decreased their invasion. Knockdown of SPARC was also observed to significantly increase the apoptosis of MGC803 and HGC 27 gastric cancer cells compared with control transfected group.

**Conclusions:**

Our data showed that downregulating of SPARC inhibits invasion and growth of human gastric cancer cells. Thus, targeting of SPARC could be an effective therapeutic approach against gastric cancer.

## Introduction

Gastric cancer is the second leading cause of cancer-related deaths worldwide and is one of the most aggressive tumors and is frequently associated with lymph node metastasis, peritoneal dissemination, and hematogenous metastasis[1]. On the whole, 65-70% of new cases and deaths from gastric cancer occur in less-developed countries[2]. In 2005, the incidence rates of gastric cancer (0.3 million deaths and 0.4 million new cases) ranked third among the most common cancers in China[3]. Current therapies for advanced stage or metastatic gastric cancer have little effect, surgical removal with resection of adjacent lymph nodes offers the only chance for cure, which is less than 33% of patients with gastric cancer. The 5-year survival rate is only 30-40%, with a poorer prognosis for advanced tumours. Understanding the molecular mechanisms underlying the progression of gastric cancer may provide insights into new therapeutic targets.

Secreted protein acidic and rich in cysteine (SPARC; also known as osteonectin or BM-40) belongs to the matricellular family of secreted proteins[4]. SPARC is a nonstructural component of extracellular matrices that modulates cell-matrix interactions, particularly during tissue development, remodeling and repair[5]. Many types of cancers are characterized by upregulated expression of SPARC[6]. Overexpression of SPARC has been documented in several types of solid tumors, such as breast[7], prostate[8], melanoma[9] and glioblastomas[10]. In contrast, lower levels of SPARC expression have been found in other types of cancers, such as ovarian[11], colorectal[12], pancreatic[13,14] and acute myelogenous leukemia[15]. These observations suggest that tumorigenic effect of SPARC is cell type specific and may be dependent of the tumor cell surrounding environment.

The knowledge about SPARC functions in gastric cancer cells is still sparse. Overexpression of the SPARC gene was observed in human gastric cancer in five other reports[16-20]. However, all above-mentioned studies had no detail in gastric cancer cell lines and carcinogenic mechanism. SPARC has been associated with aggressive stages of gastric cancer and is correlated with poor prognosis[16], which suggests that the reduction of SPARC expression may have therapeutic benefit. Indeed, expression of antisense oligonucleotides against SPARC in melanoma cells blocked tumor formation[21]. The precise biological and molecular mechanisms through which a reduction in SPARC expression might contribute to improved tumor therapy remain to be investigated. Therefore, the aim of the present study was to characterize SPARC functions in gastric cancer cells and explore its possibly carcinogenic mechanism.

## Materials and methods

### Cell culture

Human gastric cancer cell lines NCI-N87, SGC7901, MGC803, BGC823, HGC27 were obtained from the Cancer Institute of Chinese Academy of Medical Science. All cells were grown in RMPI 1640 (GIBCO™)medium supplemented with 10% fetal bovine serum, penicillin G (100 units/ml), and streptomycin (100 μg/ml) termed complete medium. Cells were maintained in monolayer culture at 37°C in humidified air with 5% CO_2_.

### Chemicals and reagents

EDTA-2 sodium, acridine orange, ethidium bromide (EB) and 3-[4,5-dimethylthiazol-2-yl]-2,5-diphenyl tetrazoliumbromide (MTT) were purchased from Sigma (St Louis, MO, USA). Mouse monoclonal antibody specific to β-actin was from Sigma. Rabbit polyclonal antibodies specific to Bcl-2 (sc-492), caspase-3 (sc-7148) and PARP (sc-7150) were bought from Santa Cruz Biotechnology (Santa Cruz, CA, USA). Mouse monoclonal antibodies specific to SPARC(sc-74295) and Bax (sc-7480) were obtained from Santa Cruz Biotechnology. Goat anti-rabbit (w3960) and anti-mouse (w3950) secondary antibodies were purchased from Promega (Madison, WI, USA).

### RNAi and transfection

Human SPARC siRNA and control siRNA were from Dharmacon Bioscience Corp (Chicago, IL, USA). Equimolar amounts of siRNAs were used as per the manufacturer's instructions with control non-targeting siRNA (CTRL). 150 000 cells were plated per six-well in DMEM with 10% FBS and were allowed to attach overnight. Equimolar amounts of siRNAs were incubated with TransIT-TKO Transfection Reagent from Mirus (Madison, WI, USA) as per the manufacturer's instructions. Cells were maintained for 48 h before experiments, unless otherwise described

### Western blot analysis

Twenty micrograms of total proteins were separated by SDS-PAGE and transferred onto a PVDF membrane. The membrane was then incubated with antibodies specific for SPARC (Santa Cruz; 1:500), or anti-β-actin (Sigma; 1:1,000) overnight at 4°C. Bound antibodies were visualized using enhanced chemiluminescence. To confirm equal loading, membranes were stripped for 30 minutes at 50°C in buffer containing 2% SDS, 62.5 mM Tris (PH 6.7), and 100 mM 2-mercaptoethanol and reprobed with an anti-β-actin antibody to demonstrate equal loading. The density of the bands was quantified by densitometric analysis using the ImageTool (version 3.0) system.

### RT-PCR

Total RNA (1-2 μg) was reverse transcribed using a SuperScript pre-amplification kit (Invitrogen, Carlsbad, CA). Primers were based on sequences reported on Genebank (NM 003118). SPARC sense sequence was 5'-GTGGGCAAAGGGAAGTAACA-3' and SPARC anti-sense sequence 5'-GGGAGGGTGAAGAAAAGGAG-3'. The expected product size of SPARC cDNA was 512bp. ß-actin sense sequence was 5'-GGCATCCTCACCCTGAAGTA-3' and ß-actin anti-sense sequence 5'-GTCAGG CAGCTCGTAGCTCT-3'. The expected product size of ß-actin cDNA was 514bp. PCR amplification was performed in 25 μl reaction volumes containing 0.2 μM dNTPs, 20 pmol of each oligonucleotide primer, and 0.2U Tag polymerase in PCR buffer. cDNA was amplified on a PCR thermal controller with an initial denaturation at 95°C for 5 min, followed by cycles of 95°C for 1 min, 65°C for 1 min, and 72°C for 1 min, 27 cycles, and a final extension step of 72°C for 10 min. The amount of starting cDNA was adjusted using β-actin intensity.

### Cell migration assay

The ability of cells to migrate through filters was measured using a BioCoat Matrigel invasion chamber (BD Biosciences, San Jose, CA). Cell culture inserts with an 8 μm pore size PET membrane were used according to the protocol of the manufacturer. The bottom chamber included medium (0.75 ml) containing 10% FCS, whereas SPARC siRNA transfected or control transfected cells (1.0 × 10^5 ^suspended in 0.5 mL of medium containing 1% FCS) were seeded into the upper chamber and incubated overnight at 37°C in a humidified atmosphere containing 5% CO_2_. Remaning cells on the upper surface were mechanically removed. Membranes were then washed, fixed, and stained by Diff-Quik (Medion Diagnostics). The number of cells that migrated to the lower surface of the filters was determined by counting stained cells under a light microscope in three independent fields (0.25 mm^2^/well).

### Cell growth and viability assay

The effect of SPARC SiRNA on the viability of cells was determined by the MTT assay. Briefly, MGC803 and HGC 27 cells were plated at 1 × 10^4 ^cells per well in ninety-six-well microtitre plates. After incubation for 72 h, cell viability was determined. Then 20 μl MTT (10 mg/ml in PBS stock, diluted to working concentration of 1 mg/ml with media) was added to each well and incubated for 4 h. After careful removal of the medium, 200 μl dimethyl sulfoxide was added to each well and shaken carefully. The absorbance was recorded on the microplate reader (ELX 800; Bio-Tek Instruments, Inc. Winooski, VT, USA) at a 570 nm wavelength. The effect of SPARC siRNA on cell growth inhibition was assessed as percentage cell viability where vehicle treated cells were taken as 100% viable.

### Cell cycle analysis and annexin V staining

For flow cytometric cell cycle analysis, the cells treated with siRNA were collected, washed with PBS, fixed in cold 70% ethanol, and stored at -20°C until staining. After fixation, the cells were washed with PBS and incubated with 50 μg ⁄mL RNaseA (Sigma) for 30 min at 37°C, before staining with 50 μg ⁄mL propidium iodide (Sigma). Apoptotic cells in early and late stages were detected using an annexin V-FITC Apoptosis Detection Kit from BioVision (Mountain View, CA, USA). In brief, the cells were transfected with siRNA. At 96 h post-transfection, culture media and cells were collected and centrifuged. After washing, cells were resuspended in 490 μL annexin V binding buffer, followed by the addition of 5 μL annexin V-FITC and 5 μL propidium iodide. The samples were incubated in the dark for 5 min at room temperature and analyzed using flow cytometry.

### Statistics

Results were expressed as mean expression levels (± SD). Student's *t*-test or rank sum test were used for statistical analysis. A p-value < 0.05 was taken as level of significance (two-sided).

## Results

### Expression of SPARC in cultured gastric cancer cells

**Figure 1:**
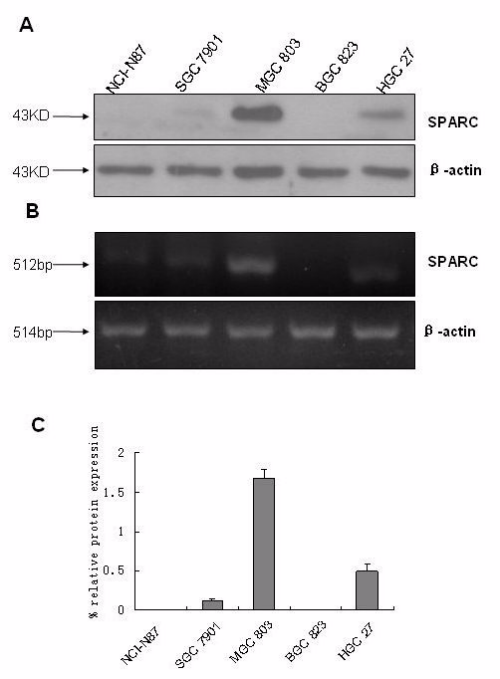
**Expression of SPARC in gastric cancer cell lines**. A, Immunoblot analysis using a rabbit polyclonal SPARC antibody (1:500). B, Specific reverse transcriptase polymerase chain reaction (RT-PCR) analysis for SPARC. β-actin was used as loading control. C, Relative SPARC mRNA expression levels. Autoradiographs were scanned and analyzed by densitometry followed by quantitation relative to β-actin. Results are shown as expression (in %) relative to β-actin and are means (± SD) of 3 experiments.

We first evaluated the endogenous expression of SPARC in several human gastric cancer cell lines. We found that SPARC protein and mRNA were prevalent in MGC803 and HGC27 cells, were produced at lower levels by SGC7901 cell line were undectable in NCI-N87 and BGC823 cell lines(Figure 1).

### Inhibition of endogenous SPARC expression

**Figure 2:**
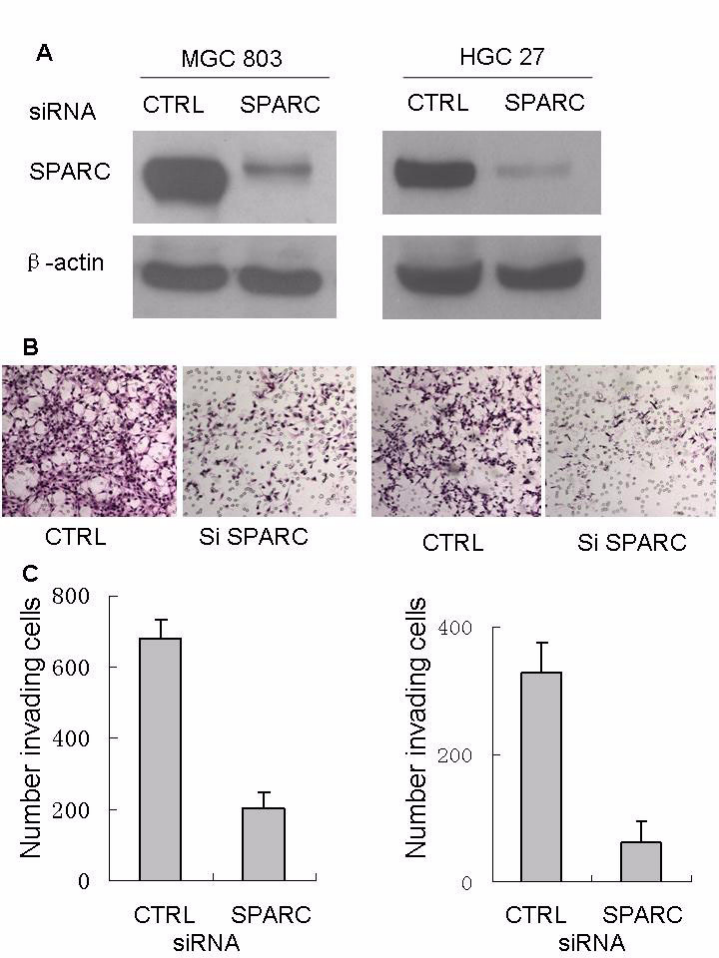
**Effect of SPARC knockdown on cell migration in gastric cancer cell lines MGC 803 and HGC 27 cells**. A. SPARC expression in MGC 803 and HGC 27, control and SPARC siRNA transfected cells was detected by immunoblot analysis using a rabbit polyclonal SPARC antibody (1:500). β-actin was used as loading control. B. Effects of SPARC knockdown on cell migration in gastric cancer cell lines. SPARC expression was knocked down in MGC 803 and HGC 27 cells using SPARC siRNA and subjected to a migration assay using a two-chambered invasion apparatus as described in Materials and Methods, histogram showing percent inhibition of MGC 803 and HGC 27 cell invasion. The experiment was done in triplicate and the value obtained from scrambled siRNA transfected cells was set as 100%.

Following this initial screening, MGC803 cells and HGC27 cells expressing relatively high endogenous SPARC were established knockdown expressing SPARC in a transient manner to determine the importance of endogenous SPARC expression. As shown in Figure 2A, SPARC expression was inhibited with SPARC siRNA transfectants in protein levels. These results suggest that these SPARC siRNAs successfully exert a silencing effect for SPARC expression.

### Downregulation of SPARC expression inhibited gastric cancer cells invasion in vitro

To determine if SPARC siRNA could reduce protumorigenic cellular behaviors associated with SPARC expression, we first determined the effect of decreased SPARC expression on tumor cell invasion. Cell invasion assay were then performed using Transwell chambers. We measured the capacity of gastric cancer cells to invade through Matrigel, an artificial extracellular matrix, after transfection with a non-targeting control siRNA or SPARC siRNA. Decreased SPARC expression led to the inhibition of invasion by 69% and 79% in MGC803 and HGC27, respectively (Figure 2B, C). Taken together, these results clearly indicate that suppression of SPARC inhibits the migration and invasion ability of MGC803 cells and HGC27 cells.

### Downregulation of SPARC expression inhibits growth of gastric cancer cells in vitro

**Figure 3:**
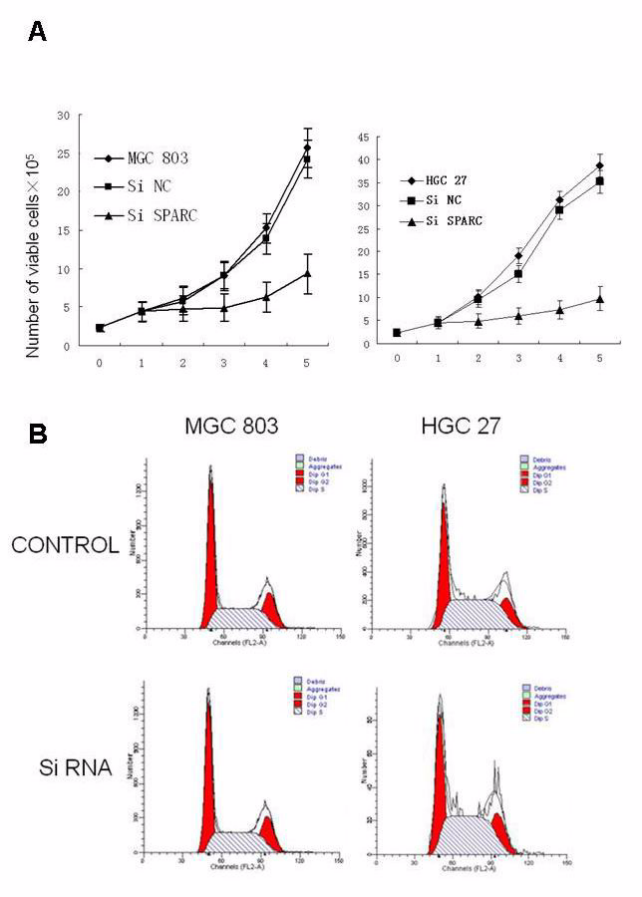
**Effects of SPARC knockdown on cell growth in gastric cancer cell lines**. the left half data represent data obtained from MGC 803 cells and the right ones represent data obtained from HGC 27 cells. A. Basal growth was determined after 48 h in complete medium by the MTT assay. Results are shown as mean growth (in %) of the respective MGC 803 and HGC 27 cell line and are means (± SE) of quadruplicate determinations from six separate experiments. Cells from the siRNA and control groups were collected for cytometry cell cycle analysis. B. Silencing of SPARC by siRNA transfection did not change cell cycle distribution in MGC 803 and HGC 27 gastric cancer cells. MGC 803 and HGC 27 cells were transfected with SPARC siRNA or negative control siRNA. At 72 h post-transfection, DNA content was measured using propidium iodide (PI) staining on flow cytometry.

We investigated whether SPARC siRNA could decrease the survival of gastric cancer cells. MGC 803 and HGC 27 gastric cancer cells transfected with SPARC siRNA survived at decreased rates relative to matched cells transfected with a non-targeting control siRNA (Figure 3A). Downregulation of SPARC expression didn't induce cell cycle arrest in gastric cancer cells. We examined the effects of SPARC siRNA on cell cycle progression. Silencing of SPARC in MGC803 and HGC27 cells didn't change G1 or S phase populations at 72 h posttransfection with SPARC siRNA in comparison with the negative control group(Figure 3B).

### Inhibition of SPARC expression enhances apoptosis in gastric cancer cells

**Figure 4:**
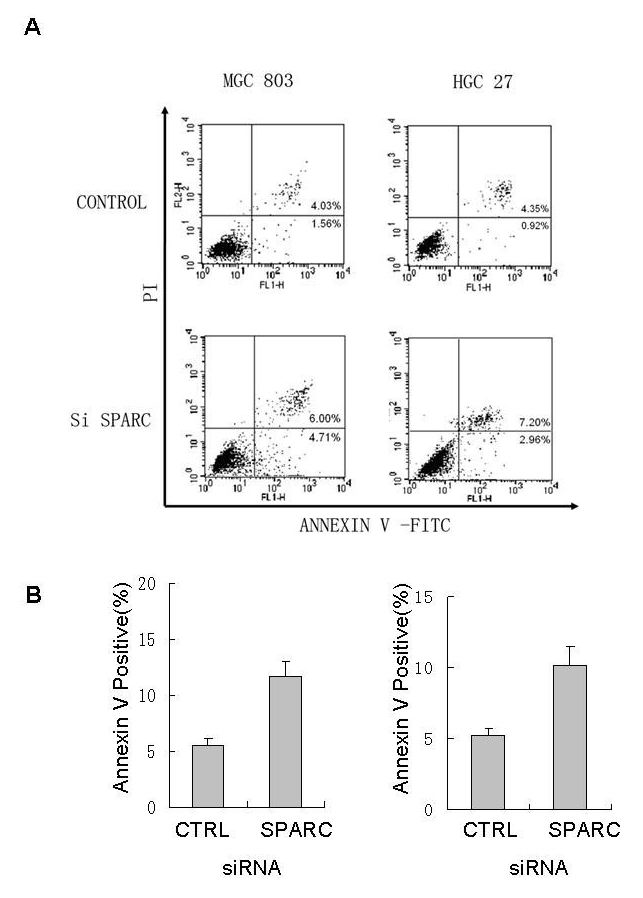
**SPARC knockdown results in induction of apoptosis in gastric cancer cell lines**. For flow cytometric analysis, cells were harvested 96 h after transfection with SPARC siRNA or negative control siRNA, then stained with annexin V-FITC and propidium iodide (PI). the left half data represent data obtained from MGC 803 cells and the right ones represent data obtained from HGC 27 cells. The percentages of annexin V/PI(early apoptotic) and annexin V/PI(late apoptotic) cells is shown in each panel. Values in bold indicate decreasing SPARC expression increased apoptosis by 65% in MGC803 and 92% in HGC27 compared with negative control siRNA.

We investigated whether SPARC siRNA could induce cell death in gastric cancer cell lines. The treatment of MGC803 and HGC27 cells with SPARC siRNA increased early apoptotic cells as well as late apoptotic cells, compared with negative control siRNA treatment (Figure 4A) as measured by the Annexin V assay. As expected, the decreased survival of the cells transfected with SPARC siRNA was associated with increased rates of apoptosis by 91% in MGC803 and 92% in HGC27 cells (Figure 4B). These findings suggest that SPARC is involved in apoptosis to maintain cellular survival in some gastric cancer cells.

### Apoptotic effect of SPARC siRNA transfected treatment in MGC 803 and HGC27 cells

**Figure 5:**
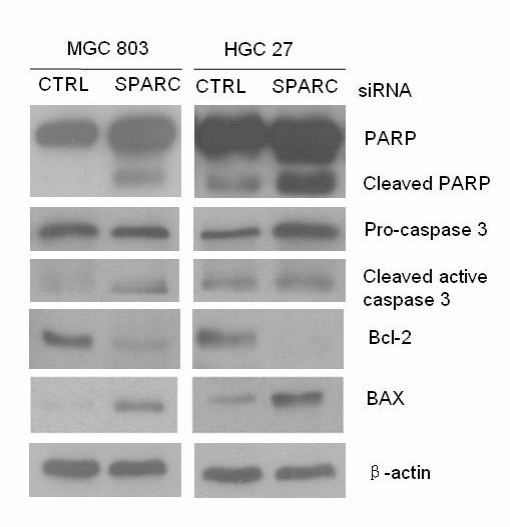
**The expression of apoptosis proteins in MGC 803 and HGC27 cells after transfection with either control or SPARC siRNA**. The cell lysates were separated on 10% SDS-PAGE gel, transferred to nitrocellulose membrane and probed with anti-PARP, anti-caspase-3, anti-Bcl-2, and anti-Bax. Protein contents were normalized by probing the same membrane with anti-β-actin. The left half data represent data obtained from MGC 803 cells and the right ones represent data obtained from HGC 27 cells.

In an effort to elucidate the mechanism of SPARC siRNA induced apoptosis in MGC 803 cells and HGC27 cells, expression levels of apoptotic-regulation proteins such as Bcl-2, Bax and caspase-3 and PARP were evaluated. MGC 803 cells and HGC27 cells were transfected with SPARC siRNA. As shown in Figure 5, There were significant differences in the expressions of Bax and Bcl-2 in MGC 803 cells and HGC27 cells in comparison with the negative control group (P < 0.05 and P < 0.01). In response to apoptotic stimuli, procaspase-3 is cleaved into a 20 kDa fragment, and the subsequent autocatalytic reaction leads to the formation of the active 17 kDa fragment. When the caspase-3 is activated, PARP is cleaved. Thus cleavage of PARP is used as an indicator of apoptosis. In order to obtain direct evidence showing the relationship of caspase activation and apoptosis, procaspase-3 cleavage and PARP were examined in MGC 803 cells and HGC27 cells after SPARC siRNA transfected. As shown in Figure 5, SPARC SiRNA induced the cleavage of 32 kDa procaspase-3 into its active 17 kDa form and cleavage of PARP appeared in MGC 803 cells and HGC27 cells.

## Discussion

Secreted protein and rich in cysteine, SPARC (also known as osteonectin; or basement-membrane-40, BM-40), is a member of a family of matricellular proteins, whose function is to modulate cell-matrix interactions and cell function without participating in the structural scaffold of the extracellular matrix. Overexpression of SPARC has been documented in several types of solid tumors, such as breast[7], prostate[8], melanoma[9] and glioblastomas[10]. In contrast, lower levels of SPARC expression have been found in other types of cancers, such as ovarian[11], colorectal[12], pancreatic[13,14] and acute myelogenous leukemia[15]. These observations suggest that tumorigenic effect of SPARC is cell type specific and may be dependent of the tumor cell surrounding environment.

The knowledge about SPARC functions in gastric cancer cells is still sparse. Some immunohistochemical studies[16-20,22] collectively reported an up-regulation of SPARC in gastric cancer compared with nonneoplastic mucosa. Wewer et al.[17] described a differential expression of SPARC in the epithelial and stromal compartments of six gastric cancer specimens. Maeng[18] found that SPARC is highly expressed in reactive stroma associated with invasive differentiated adenocarcinomas and that it may serve as a useful clinical diagnostic marker for stomach cancer. Wang et al.[16] also found a differentially expressed SPARC in gastric cancer patients as assessed by gene array analysis, quantitative RT-PCR, and immunostaining, higher SPARC expression was significantly associated with tumour progression and the advanced stages of gastric cancer. Franke et al.[20] demonstrated on a larger patient series that SPARC is differentially expressed in gastric cancers and that its expression correlates with tumor progression and nodal spread using tissue microarrays (TMAs), The level of expression of SPARC, determined by immunohistochemistry, correlated in intestinal-type gastric cancer with the local tumor growth, nodal spread, and tumor stage according to the International Union Against Cancer. Zhao ZS et al.[19] found that SPARC was detected in 334 of 436 human gastric cancer cases and was highly expressed in 239 tumors. In stages I, II, and III, the 5-year survival rate of patients with a high expression of SPARC was significantly lower than those in patients with low expression. Further multivariate analysis suggested that upregulation of SPARC, MMP-2, and integrin beta1, were independent prognostic indicators for the disease.

We have Collected 49 gastric cancer tissues and corresponding normal tissues through surgical procedures(Jie Yin, Guowei Chen, Si Liu, Jianxun Zhao, Yucun Liu: Expression of SPARC in human gastric cancer is associated with the clinical-pathological features, submitted). The distribution and expression of SPARC were observed by immunohistochemistry, Western Blotting and RT-PCR, respectively. SPARC protein and mRNA expressed significantly higher in gastric cancer tissues compared to normal tissues. The degrees of its expression were associated with differentiation of tumor, TNM division, peritoneal seeding and vascular invasion remarkably. Patients with high expression of SPARC have worse prognosis than those with low expression of SPARC.

Taken together, higher SPARC expression was significantly associated with tumour progression and advanced stages of gastric cancer. Recent research of Inoue M et al[23] even identifed SPARC as a candidate target antigen for immunotherapy of various cancers including gastric cancer by genome-wide cDNA microarray. It is exciting that therapy targeting the SPARC subunit may be a useful approach to suppress gastric cancer growth. However, the molecular mechanisms responsible for the oncogenesis of SPARC in gastric cancer is not entirely understood. Through expression analysis of a panel of gastric cancer cell lines, we showed that SPARC is also overexpressed in sevel human gastric cancer cell lines. Therefore, we tested our hypotheses that SPARC may be a key molecule in gastric cancer invasion, and that targeting SPARC may present a novel therapeutic strategy for anti-invasion of gastric cancer.

Dissemination of cancer cells, either locally or at distant metastatic sites, requires that malignant cells acquire the ability to invade the basement membrane and to adhere to other matrices. It has been suggested that SPARC may play a key role during the initial steps in the process of tumour invasion and metastasis[24]. In addition, SPARC can induce the expression of metalloproteinases or enzymes that subsequently play an important role in the degradation of basal membranes and extracellular matrix components[25]. SPARC was associated with the invasiveness of meningiomas[26,27] and gliomas[28]. Furthermore, suppression of SPARC expression using antisense RNA inhibited motility and invasion of human breast cancer cells in vitro[21].

To determine if SPARC siRNA could reduce protumorigenic cellular behaviors associated with SPARC expression, we first determined the effect of decreased SPARC expression on tumor cell invasion. We measured the capacity of gastric cancer cells to invade through Matrigel, an artificial extracellular matrix, after transfection with SPARC siRNA or a non-targeting control siRNA. Decreased SPARC expression led to the inhibition of invasion by 69% and 79% in MGC803 and HGC27, respectively. Thus, SPARC siRNA can decrease gastric cancer invasion in vitro.

A recent study found that SPARC protects cells from stress-induced apoptosis in vitro through an interaction with integrin β1 heterodimers that enhance ILK activation and prosurvival activity[28]. Initial studies using antisense RNA strategies completely abrogated human melanoma growth in nude mice[21]. Horie et al.[29] showed that the downregulation of SPARC expression induced growth inhibition with G_1 _arrest in human melanoma cells. It has been reported that SPARC promotes glioma cell survival through Akt activation through integrin signaling under serum-free conditions [30]. These reports strongly suggest that SPARC plays a role as an antistress factor.

On the other hand, some articles found that SPARC may promote apoptosis in cancer cells. Yiu and colleagues[11] showed that exogenous treatment of various ovarian cancer cell lines with SPARC induced apoptosis. Said and Motamed[31] found SPARC exposure increased cleaved caspase 3 in human ovarian carcinoma cells which supported the former observation. Pancreatic[13] and ovarian cancers[30] exhibited greater growth and reduced apoptosis when implanted in SPARC^-/-^. In colorectal cancer cell lines, overexpression of SPARC reduced cell viability and enhanced apoptosis in cells exposed to various chemotherapeutic agents[32].

These seemingly paradoxical observations within each type of cancer and across different cancers can be explained by Tai's understanding of SPARC biology[33]: smaller peptide fragments of SPARC representing the different domains of SPARC confer biological activities which at times, oppose those of other fragments or the native SPARC protein. Since the protease profile of the tumor microenvironment may differ in different types of cancers, and as SPARC is known to undergo proteolysis by matrix metalloproteinases[34], these differences, in combination with changes in the local composition of matrix molecules and cytokines, may all be contributing to the complex behavior of SPARC in different types of cancer.

To elucidate the effects of SPARC siRNA on gastric cancer cell growth, MTT proliferation assay was performed to compare the proliferation between SPARC siRNA transfected and control transfected MGC803 and HGC 27 cells. MGC803 and HGC27 gastric cancer cells transfected with SPARC siRNA survived at decreased rates relative to matched cells transfected with a non-targeting control siRNA (Figure 3). The decreased survival of the cells transfected with SPARC siRNA was associated with increased rates of apoptosis as measured by the Annexin V assay. Decreasing SPARC expression increased apoptosis by 91% in MGC803 and 92% in HGC27 (Figure 4B).

Active caspases play an important role in the induction of apoptosis. When caspase-3 was activated, PARP is cleaved late. Usually the cleavage of PARP was used as an indicator of apoptosis. In the present study, we found SPARC siRNA activated caspase-3 to produce cleaved caspase-3 (p17) fragments in MGC 803 cells and HGC 27 at 48 h. At the same time, the cleavage of PARP was also detected. The results indicate that SPARC induced fragmentation of PARP as well as increased caspase-3 activity in MGC 803 cells.

The Bcl-2 family proteins have been reported to regulate apoptosis by controlling the mitochondrial membrane permeability. SPARC up regulated the expression of Bax and down regulated the expression of Bcl-2 in MGC 803 cells and HGC 27 cells. We found that SPARC siRNA could induce gastric cancer cell apoptosis and simultaneously reduce the ratio of Bcl-2 to Bax. Therefore, the regulation of Bcl-2 and Bax expression may be a key mechanism underlying SPARC induction of apoptosis in gastric cancer cells.

So our data indicated that downregulation of SPARC inhibited cell proliferation of gastric cancer cells by apoptosis initiation, which conscience with melanoma and glioma, but contrary to ovarian and pancreatic cancer. The induction of apoptosis was partly regulated to mitochondrial pathway such as activation caspase pathway as well as cleavage of PARP. Future study needs to focus on the exact mechanism.

In conclusion, our current data suggested that SPARC played important roles in apoptosis and metastasis of gastric cancer. At present, there are no effective approaches for curing late stage gastric cancer. As elevated SPARC expression is associated with decreased gastric cancer patient survival[16], we believe that our results, demonstrating decreased invasion and increased cell death with siRNA directed against SPARC, suggest that decreasing SPARC expression may have therapeutic benefit for gastric cancer patients.

## Competing interests

The authors declare that they have no competing interests.

## Authors' contributions

JY and GWC participated in study design, carried out most of the experiments, and drafted the manuscript. YCL designed the study, wrote the manuscript. PYW, HQG and JZ conceived of the study, and participated in its design and performed the statistical analysis. SL and JZ assisted with cell culture. YLW and XW assisted with the critical revision of the manuscript. All authors read and approved the final manuscript.
